# Investigating the professional capability of triage nurses in the emergency department and its determinants: a multicenter cross-sectional study in Iran

**DOI:** 10.1186/s12873-023-00809-7

**Published:** 2023-04-01

**Authors:** Maryam Aghabarary, Zahra Pourghaedi, Mostafa Bijani

**Affiliations:** 1 Social Determinants of Health Research Center, Alborz University of Medical Sciences, Karaj, Iran; 2 Student Research Committee, Alborz University of Medical Sciences, Karaj, Iran; 3 Department of Medical Surgical Nursing, School of Nursing, Fasa University of Medical Sciences, Fasa, Iran

**Keywords:** Capability, Triage, Nurses, Emergency Department

## Abstract

**Background:**

Planning to improve the professional capabilities of triage nurses requires determining the level of professional capabilities and its determinants. In this regard, the present study was conducted to determine the professional capability of triage nurses and its determinants for the first time in Iran.

**Methods:**

A descriptive cross-sectional multicenter study was conducted in 2022. The research population included all nurses working in triage units of emergency departments of seven selected hospitals in Fars Province, south of Iran. The samples were selected using convenience sampling. The data collection tools were the “Triage nurses’ professional capability questionnaire in the emergency department” and a questionnaire to investigate determinants of triage nurses’ professional capability. Descriptive and analytical (Pearson’s correlation test and multiple linear regression analysis) were used for data analysis in the SPSS software version 27. P values ≤ 0.05 were considered significant.

**Results:**

Out of 580 participants, 342 (59%) were female. The professional capability of triage nurses was at a moderate level with a mean score of 124.11 ± 14.72. The mean score of clinical competence, psychological empowerment, and professional commitment was 71.56 ± 9.67, 19.86 ± 3.95, and 32.69 ± 3.54, respectively. The results of multiple linear regression analysis showed that 5 factors, including participation in educational courses (p < 0.001), having clinical experience and specialized knowledge in emergency department (p < 0.001), error registry and assessment system (p < 0.001), managers’ support (p < 0.001), and recruiting experienced staff (p = 0.018) affected the nurses’ professional capability.

**Conclusion:**

In the present study, the triage nurses had moderate levels of professional capability. It is necessary that nursing managers develop effective plans to improve the professional capability of triage nurses in emergency departments to enhance the quality and effectiveness of emergency services.

**Supplementary Information:**

The online version contains supplementary material available at 10.1186/s12873-023-00809-7.

## Introduction

The emergency department (ED) is one of the most important and sensitive hospital departments that plays a critical role in offering emergency care services to patients [1]. During the 1950s and in the early 1960s, with an increase in the number of patients presenting to EDs, the authorities felt a need to separate the patients with urgent medical needs from those with less serious problems [2]. Therefore, triage was suggested to prioritize patients in ED [3]. Triage is the process of prioritizing patients for healthcare services based on the severity of the diseases or injury to perform the best treatment intervention for most people in the shortest possible time [4]. Today, triage is an inseparable part of emergency management in hospitals and an important indicator in assessment and accreditation of ED services [5]; since the accuracy of triage decisions can affect ED achievements [6]. Accurate and quick triage of the patients is a key factor in the successful performance of emergency departments. If triage is done without due skills and knowledge, the patients are misclassified [7]. In other words, the effectiveness of triage systems lies in the professional knowledge and clinical skills of the ED staff, including nurses. The nurses are the main triage operators in EDs and play an important role in prioritizing the needs of the patients that are in critical conditions [8]. The Emergency Nurses Association (ENA) states that triage nurses should have capabilities like professional knowledge, clinical experience, communication skills, critical thinking, patient’s health assessment skills, clinical decision-making, teamwork, and mental and physical capability to work in stressful conditions to have an effective function [9]. Therefore, triage nurses have a crucial role in determining and prioritizing the needs of the patients with life-threatening conditions requiring immediate attention. Hence, it is important to pay attention to their professional capability for optimal care delivery [10].

Professional capability is a broad concept that can be defined according to context, attributes, position, and perspective of people [11]. According to a study by Bijani et al. in 2018, in order for triage nurses to have professional capability, they should not only have clinical competence, but possess psychological skills and professional commitment. In other words, the triage nurses’ professional capability is comprised of three components, including clinical competence, psychological empowerment, and professional commitment [12]. If triage nurses lack the required professional capabilities, it would result in errors in triage and problems such as increased length of hospital stay, delay in transferring patients from ED to other wards, ED crowding, reduced patient satisfaction, reduced quality of care, worsening of the patients’ conditions, and sometimes permanent injury to the patients and even death [13]. Although nurses play a part in the occurrence of these triage errors, they are not solely responsible for them.

In other words, if these skills are not practiced at a standard level, it will endanger the outcomes of clinical care and the effectiveness of emergency departments. Despite the importance of the role of standard triage and the triage nurses’ professional capability in saving lives, no study has directly evaluated this concept; however, the results of the studies that assessed the professional knowledge and clinical skills of the nurses showed a moderate level of clinical skills [11]. Studies conducted in the world and Iran suggest that there are serious concerns in these areas so that lack of professional knowledge and clinical skills in triage nurses has received attention as a global problem [14]. According to a study in Addis Ababa, Ethiopia in 2018, more than half of the participants had moderate levels of triage skill and its subdomains [6]. In 2019, Duko et al. conducted a study in Hawassa, Ethiopia and reported the low level of triage knowledge among nurses. They suggested that the hospitals and ministry of health should provide training to improve the nurses’ triage knowledge and skills [15]. The results of a study by Phukubye et al. (2016) in Limpopo Province, South Africa showed that despite having adequate triage knowledge, the nurses’ triage practice was poor since they obtained low scores in response to questions related to triage practice [8]. In Turkey, Aslanoglu and Ayyildiz (2021) reported the moderate triage knowledge level of the nurses [16]. The triage knowledge of the nurses was also low in a study by Bista et al. (2022) in Kathmandu, Nepal [17]. Moreover, the results of a study by Twagirayezu et al. (2021) in Rwanda showed the inadequate level of triage knowledge and skills among nurses [18].

Studies conducted in Iran suggest that patient triage is done by nurses that are not adequately knowledgeable. Mirhaghi and Roudbari (2011) conducted a study to determine triage quality in Sistan & Baluchestan hospitals and found that triage nurses misclassified 15% of the patients in under triage and 48% in over triage levels due to lack of triage knowledge and skills [19]. Haghigh et al. (2017) reported the undesirable awareness level of the emergency department nurses involved in patient triage in Ahvaz educational hospitals [1]. The results of a recent systematic review by Javadi et al. (2021) revealed the overall moderate triage skill level of the nurses in Iran [11].

## Background in Iran

In view of shortage of equipment and physical space and overcrowding in the emergency departments of hospitals in Iran, in May 2010, the healthcare branch of the Ministry of Health mandated that all emergency departments must be equipped with a triage unit and stressed that triage should be regarded as an important criterion in evaluation of the effectiveness and efficiency of emergency services provided by hospitals. In the emergency departments of hospitals in Iran, patients are triaged according to a five-level algorithm defined by Emergency Severity Index (ESI). ESI triage algorithm is a method of hospital triage which assigns patients to one of five levels (level 1 most urgent to level 5 least urgent).

Triage nurses must have a minimum of a bachelor’s degree in nursing and one year’s experience of practice. In every shift in a triage unit, two triage nurses must be present and prioritize patients according to the five-level ESI triage algorithm. Triage personnel work rotating shifts which are divided into morning, afternoon, and night shifts. All patients with any kind of clinical issue and of any age who visit or are referred to an emergency department are initially examined by a triage nurse and then examined by the physician or emergency medicine doctor of the department for more accurate prioritization.

In addition to research results, the clinical experience of the authors suggests that the professional capability of triage nurses is not measured upon recruitment in many Iranian hospitals. This is while recruiting nurses that lack the required triage capabilities would definitely lead to errors in triage of the patients and worsening of their condition. Therefore, it is necessary to improve the effectiveness of hospital EDs through attending to patients’ needs in a timely manner, prevention of complications and mortality, increasing patient satisfaction, and careful planning to enhance the professional capabilities of triage nurses. For this purpose, it is necessary to measure the professional capability of triage nurses, determine its associated factors and then design and implement appropriate plans to improve it. In this regard, the present study was conducted to determine the professional capability of triage nurses and its determinants for the first time in Iran.

## Methods

### Study design, setting, and participants

A descriptive cross-sectional multicenter study was conducted in 2022. The research population included all nurses working in triage units of EDs of seven selected hospitals (two hospitals in Shiraz, one hospital in Fasa, one hospital in Jahrom, one hospital in Darab, one hospital in Larestan, and one hospital in Gerash) in Fars Province, south of Iran (n = 778).The samples were selected using convenience sampling method according to the inclusion criteria. The inclusion criteria were a bachelor’s degree or higher in nursing, a minimum of 6 months of experience in the ED triage unit, working full-time in the ED triage unit, and willingness to participate in the study.

### Evaluation tool and data collection

Two questionnaires were used for data collection in the present study, including the “Triage nurses’ professional capability questionnaire in the emergency department” and a questionnaire to investigate determinants of triage nurses’ professional capability along with a demographics sections.

#### A. demographics section

Demographic characteristics including age, sex, marital status, education level, work experience, shift, hospital, and interest in working as a triage nurses were collected.

#### B. Triage nurses’ professional capability questionnaire in the emergency department

This questionnaire includes 35 items in 3 domains, including clinical competence (items 1–20), psychological empowerment (item 21–26), and professional commitment (items 27–35). This questionnaire was designed and validated by Bijani et al. in 2018 as a nursing PhD thesis. The items are rated in 5-point Likert scale from 1 (not important at all) to 5 (very important) as a self-report instrument. The total score of the questionnaire ranges between 35 and 175, and scores of 35–81, 82–128, and 129–175 are considered as poor, moderate, and good professional capability, respectively. Moreover, the score of each domain can be calculated separately as clinical competence (1-100), psychological empowerment (1–30), and professional commitment (1–45). The internal consistency reliability of the questionnaire (Cronbach’s alpha) was 0.89 for the whole scale and 0.92 for clinical competence, 0.87 for psychological empowerment, and 0.89 for professional commitment domains. The interclass correlation coefficient (ICC) of the whole instrument was 0.90 [20]. In the present study, the internal consistency reliability of the questionnaire (Cronbach’s alpha) was 0.95 for the whole scale and 0.94 for clinical competence, 0.93 for psychological empowerment, and 0.79 for professional commitment domains.

#### C. Triage nurses’ professional capability determinants questionnaire

A 10-item researcher-made questionnaire in a 5-point Likert scale from 1 (very low) to 5 (very high) was used. Based on weighing of significance, each item is given a minimum score of 1 and a maximum score of 5. Thus, the score for each item was reported based on the mean and standard deviation of the highest possible score, i.e. 5, and the items which earned higher mean scores were placed in upper levels (Table 1). Considering the objectives of the study, the scale was drafted based on a literature review and the results of qualitative studies conducted by Bijani et al. (2019) and Najafi et al. (2021) [21, 22]. In order to assess the validity of the questionnaire content validity were used. In order to investigate content validity, Content Validity Ratio (CVR), and Content Validity Index (CVI) were used. The necessity of the items was determined by the experts as ‘necessary’, ‘useful but not necessary’, and ‘not necessary’ considering CVR. In doing so, we collected 15 experts (8 nursing faculty members with experience in triage and 7 emergency medicine specialists) opinions and values greater than 0.49 were considered acceptable based on the Lawshe Table  [23]. The CVR of 10 items of was higher than 0.49. Regarding CVI, the 15 experts were requested to evaluate the items in terms of relevance, clarity, and simplicity. In this respect, scores above 0.79 were considered acceptable. Based on the results the CVI of 10 items of was higher than 0.79. Finally, the reliability of the questionnaire was assessed using the test-retest method. In doing so, the questionnaire was given to 100 triage nurses in two stages with a two-week interval. The intra-class correlation coefficient (ICC) across the 10 item was 0.79, which indicates the appropriate internal consistency of this questionnaire.

**Table 1:** Mean and standard deviation of professional capability determinants questionnaire items (organized from highest to lowest mean)

	Items	Mean (SD)
professional capability determinants	Recruiting experienced staff	3.78 ± (0.65)
	Having clinical and specialized experience in emergency department	3.47 ± (0.63)
	Participation in educational workshops or refresher courses on triage	3.32 ± (0.56)
	Standard physical environment for triage	3.19 ± (0.66)
	Familiarity with triage guidelines	3.19 ± (0.60)
	Continuous monitoring and evaluation of triage nurses’ clinical practice by managers	3.18 ± (0.63)
	Availability of formulated guidelines regarding the job description and responsibilities of triage nurses	3.15 ± (0.64)
	Planning and monitoring for evaluating, determining, and removing error causes (error registry and assessment system) in patients triage	2.94 ± (0.68)
	Personal and professional conflicts between triage doctors and nurses	2.89 ± (0.69)
	Managers’ support (encouragement and motivation)	1.80 ± (0.69)

After receiving permission to enter the research setting (triage units of selected hospitals) data collection was done through distributing the questionnaires (January - August 2022). For this purpose, a number of liaisons were selected in each hospital to distribute the paper of questionnaires. The second author and liaisons gave the questionnaires to eligible nurses. After explaining the methodology and objectives of the study, the participants were asked to complete the questionnaires.

### Ethical considerations

This study was part of an MSc thesis in nursing approved by the School of Nursing, Alborz University of Medical Sciences. Ethical clearance was obtained from the Ethics Committee of Alborz University of Medical Sciences (IR.ABZUMS.REC.1401.007). Participation was voluntary and returning the completed questionnaires was considered as consent to participation. The questionnaires were anonymous and the participants’ data were confidential.

### Statistical analysis

After data collection, the second author (ZP) of this article performed the data entry. Statistical analysis was performed by a statistician outside the research team.The data were analyzed using descriptive and analytical statistics. First, the demographic characteristics of the research population were determined, and the main variables were examined using mean and standard deviation. According to Kolmogorov-Smirnov normality test research variables had a normal distribution. So, Pearson’s correlation test was used to examine the relationship between professional capability determinants and professional capability domains. Multiple linear regression models were used to predict the professional capability according to its determinants. The regression test was performed using the simultaneously method (Enter). The SPSS software version 27 was used for data analysis. P ≤ 0.05 were considered significant.

## Results

Out of the 778 triage nurses, 685 met the inclusion criteria and were invited to join the study, of whom 580 returned their questionnaires (response rate = 84.67%). Of 580 participants, 342 (59%) were female and 550 (94.8%) had a bachelor’s degree in nursing. The mean age of the participants was 31.36 ± 5.02 years (range: 22–47 years) and the mean work experience as a triage nurse was 4.95 ± 4.25 years. Other demographic characteristics are presented in (Supplementary file 1).

Table 2 shows the mean and qualitative classification of the triage nurses’ professional capability. The mean score of the triage nurses’ professional capability, clinical competence, psychological empowerment, and professional commitment was 124.11 ± 14.72, 71.56 ± 9.67, 19.86 ± 3.95, and 32.69 ± 3.54, respectively. According to Table 2, more than 50% of the nurses had moderate levels of professional capability, clinical competence, psychological empowerment, and professional commitment.

**Table 2:** Mean, standard deviation, and qualitative classification of nurses’ professional capability

Professional capability and its domains	Qualitative classification	Score range	Number (%)	Mean (SD) (Total)	Range (lowest-highest)
Clinical Competence	Poor	20–47	7 (1.2%)	71.56 ± (9.67)	40–100
	Moderate	48–73	318 (54.8%)		
	Good	74–100	255 (44%)		
Psychological Empowerment	Poor	6–14	67 (11.6%)	19.86 ± (3.95)	11–30
	Moderate	15–22	328 (56.6%)		
	Good	23–30	185 (31.9%)		
Professional Commitment	Poor	9–21	1 (0.2%)	32.69 ± (3.54)	21–44
	Moderate	22–34	405 (69.8%)		
	Good	35–45	174 (30%)		
Professional capability	Poor	35–81	0 (0%)	124.11 ± (14.72)	82–173
	Moderate	82–128	349 (60.2%)		
	Good	129–175	231 (39.8%)		

Table 1 shows the frequency percentages and mean scores of the determinants of professional capability. Recruiting experienced staff with a mean score of 3.78 and having clinical experience and specialized knowledge in the emergency department with a mean score of 3.47 had the highest mean scores among the determinants of triage nurses’ professional capability (Table 1).

Table 3 presents the results of Pearson’s correlation coefficient between the determinants of professional capability and its domains. According to the results, except for doctor-nurse conflicts (p > 0.05), other determinants had significant positive correlation with triage nurses’ professional capability (p < 0.05). Having clinical experience and specialized knowledge with a correlation coefficient of 0.41 followed by participation in educational courses and error registry and assessment system with correlation coefficients of 0.38 had the strongest correlation with professional capability (Table 3).

**Table 3:** Correlation between professional capability determinants and professional capability domains

Variables	Clinical Competence		Psychological Empowerment		Professional Commitment		professional capability (Total)	
	r	P- value	r	P- value	r	P- value	r	P- value
**Participation in educational courses**	0.34	< 0.001	0.30	< 0.001	0.29	< 0.001	0.38	< 0.001
**Having experience and knowledge**	0.38	< 0.001	0.33	< 0.001	0.32	< 0.001	0.41	< 0.001
**Standard physical environment**	0.27	< 0.001	0.24	< 0.001	0.24	< 0.001	0.30	< 0.001
**Evaluation by managers**	0.35	< 0.001	0.27	< 0.001	0.25	< 0.001	0.36	< 0.001
**Familiarity with guidelines**	0.33	< 0.001	0.30	< 0.001	0.28	< 0.001	0.36	< 0.001
**Availability of formulated guidelines**	0.34	< 0.001	0.29	< 0.001	0.30	< 0.001	0.37	< 0.001
**Error registry and assessment system**	0.35	< 0.001	0.32	< 0.001	0.28	< 0.001	0.38	< 0.001
**Managers’ support**	0.26	< 0.001	0.25	< 0.001	0.03	0.539	0.24	< 0.001
**Recruiting experienced staff**	0.27	< 0.001	0.18	< 0.001	0.30	< 0.001	0.30	< 0.001
**Doctor-nurse conflicts**	-0.06	0.140	-0.07	0.093	0.03	0.456	-0.05	0.215

**r: Pearson’s correlation coefficient**

In the present study, multiple regression analysis was used to predict the nurses’ professional capability according to its determinants. According to regression model assumptions, ANOVA, and coefficient of determination (R^2^), the F-statistic value was 29.45 (p < 0.05), indicating that the regression model was appropriate, and predictor variables could significantly predict the criterion variable (professional capability). The coefficient of determination (R^2^) is the variance of the criterion variable predicted by predictor variables. The modified R2 value was 0.329 indicating that predictor variables of model (determinants) could predict 32.9% of the variance or changes of the nurses’ professional capability. The results of multiple regression analysis confirmed the predictive role of 5 factors; therefore, it could be concluded that 5 factors (participation in educational courses, having clinical experience and specialized knowledge in emergency department, error registry and assessment system, managers’ support, and recruiting experienced staff) affected the nurses’ professional capability (p < 0.05). These factors had a positive correlation with professional capability indicating that an improvement in these factors improved the nurses’ professional capability. The standard coefficients of participation in educational courses, having clinical experience and specialized knowledge in emergency department, error registry and assessment system, managers’ support, and recruiting experienced staff were 0.180, 0.252, 0.169, 0.129, and 0.089, respectively. In conclusion, these factors could predict the nurses’ professional capability (Table 4).

**Table 4:** Prediction of professional capability according to its determinants using multiple regression analysis

Predictor variables	Unstandardized coefficient	Standard error	Standardized coefficient	T- value	**P- value	Collinearity Statistics	
						*VIF	**Tolerance**
Constant (c)	**60.35**	**4.71**	**–**	**12.80**	**< 0.001**	**–**	**–**
Participation in educational courses	4.72	1/00	0.180	4.71	< 0.001	1.26	0.791
Having experience and knowledge	5.89	0.907	0.252	6.49	< 0.001	1.31	0.766
Standard physical environment	-2.07	1.11	-0.092	-1.87	0.062	2.12	0.472
Evaluation by managers	1.07	1.29	0.046	0.832	0.406	2.61	0.384
Familiarity with guidelines	1.74	1.35	0.071	1.29	0.197	2.0	0.384
Availability of formulated guidelines	1.75	1.20	0.076	1.46	0.146	2.33	0.430
Error registry and assessment system	3.67	0.957	0.169	3.84	< 0.001	1.68	0.596
Managers’ support	2.73	0.757	0.129	3.60	< 0.001	1.10	0.906
Recruiting experienced staff	2.02	0.849	0.089	2.37	0.018	1.22	0.818
Doctor-nurse conflicts	-1.21	0.729	-0.057	-1.67	0.096	1.02	0.980

**Multiple linear regression model (simultaneous (Enter) method)**

* Variance inflation factor (VIF) cutoff value: 5

** P ≤ 0.05 were considered significant

## Discussion

The present study was conducted to determine the triage nurses’ professional capability and its determinants in EDs of selected hospitals in Fars Province, south of Iran. According to the results, more than half of the nurses had moderate levels of professional capability. Evaluation of clinical competence, as a domain of professional capability encompassing professional knowledge, clinical skill, and clinical judgment, showed that more than 50% of the nurses had moderate levels of clinical competence. Kerie et al. (2018) and Al-Rawee et al. (2022) also reported moderate levels of clinical competence in triage nurses in terms of professional knowledge and clinical skill [6, 24]. This is while the results of studies by Duko et al. (2019), Twagirayezu et al. (2021), Haghdoust et al. (2011), and Haghigh et al. (2017) showed that emergency nurses had inadequate/low levels of triage knowledge and skills [1, 15, 18, 25].

Since triage nurses had weak/moderate levels of clinical competence in the present and previous studies, especially in the areas of clinical skill and professional knowledge, nursing managers should adopt proper measures to improve these areas to enhance the quality of patient triage and effectiveness of emergency services. In this regard, Rahmati et al. (2013) found that holding training courses for triage nurses was effective in improving their professional knowledge and clinical skill and increasing the patients’ satisfaction with the quality of emergency care services [26].

One of the important aspects of the present study was evaluation of the triage nurses’ clinical judgment skills as an important area of clinical competence. Contrary to previous study, clinical judgment was considered as an important component of clinical competence in the present study since according to a study by Kantar and Alexander (2012), clinical judgment is a key skill for all nurses, especially emergency nurses, and plays an important role in correct identification of the patients’ problems and clinical decision-making [27]. Therefore, it is necessary that nursing managers, in addition to professional knowledge and clinical skill, pay special attention to improving the clinical judgment skills of triage nurses.

Psychological empowerment encompasses resilience, emotional stability, and self-confidence. The results showed moderate levels of psychological empowerment in more than half of the nurses. Eghbali and Najafi (2020) also found moderate levels of resilience and emotional stability in emergency nurses [28]. In 2019, Bijani et al. conducted a study to determine the challenges and determinants of the quality of triage in the south of Iran and found that psychological empowerment including resilience and emotional stability were the most important determinants of the quality and effectiveness of triage by emergency nurses. It is necessary that triage nurses have high levels of resilience to cope with difficult, stressful, and unpredictable conditions, overcome the emotional and physical fatigue resulting from the work environment, and maintain their psychological well-being [21]. The results of a study by Lin et al. (2019) showed that emergency nurses should be resilient to perform effective clinical interventions in critical and emergency situations [29]. Hence, considering the complex, stressful, and unpredictable situations of the emergency departments, it is necessary that senior managers of emergency wards design training courses to improve the nurses’ psychological skills. In this regard, Ahmadi et al. (2019) found that using training programs aiming at enhancing the nurses’ resilience had a significant positive association with improved professional quality of life and increased patient satisfaction [30].

Professional commitment encompasses three subdomains, including personal development, adherence to ethical principles, and observance of the principles of communication. The results of the present study showed moderate levels of professional commitment in the participants.

The scores of the areas of treating patients with dignity, respecting their privacy, observing justice in triage, responsibility and responsiveness, and maintaining a respectful communication were above average. In one study, Bijani et al. (2018) reported that observing justice in triage, having respect for patient’s dignity and privacy, and establishing a respectful communication were the most important professional commitments from the participants’ perspectives [12].

The results showed that 5 factors, including participation in educational courses, having clinical experience and specialized knowledge in emergency department, error registry and assessment system, managers’ support, and recruiting experienced staff affected the nurses’ professional capability. Among these factors, recruiting experienced staff and having clinical experience and specialized knowledge in emergency department were the most important determinants of the triage nurses’ professional capability. Moreover, having clinical experience and specialized knowledge followed by participation in educational courses and error registry and assessment system had the strongest correlation with professional capability. In this regard, the results of a study by Bijani and Khaleghi (2019) showed that having clinical knowledge, efforts for continuous personal development and having up-to-date knowledge, work experience, and recruiting experienced and skilled personnel were the most important determinants of triage quality from the perspectives of triage nurses [21]. Najafi et al. (2021) also found that having professional skills and clinical knowledge and recruiting experienced doctors and nurses in triage were important determinants of triage quality [22]. Aloyce et al. (2014) reported that most of the triage nurses misclassified the patients due to deficits in knowledge and skills regarding triage [31]. Widgren (2012) stated that having knowledge about triage protocols, diseases, and emergency conditions is a key factor in the triage nurses’ success in correct classification of the patients and high-quality and effective triage [32].

### Strengths of the study

This study was the first Iranian multicenter study of the triage nurses’ professional capability and its determinants in the EDs of selected hospitals using a comprehensive standard questionnaire. Another strong point of the present study was the use of multiple regression analysis to predict the determinants of professional capability.

### Study limitation

This study was carried out in the EDs of selected hospitals in Fars province in the south of Iran, its results may not be easily extrapolated to other regions in Iran and other countries. So, it is recommended that similar studies be conducted in other Iranian cities and other countries to compare the results.

## Conclusion

In the present study, the triage nurses had moderate levels of professional capability. It is necessary that nursing managers develop effective plans to improve the professional capability of triage nurses in emergency departments to enhance the quality and effectiveness of emergency services.

## Electronic supplementary material

Below is the link to the electronic supplementary material.

Supplementary Material 1

## Data Availability

The datasets generated and/or analysed during the current study are not publicly available due to the necessity to ensure participant confidentiality policies and laws of the country but are available from the corresponding author on reasonable request.

## Abbreviations

ED: Emergency department

## References

[1] Haghigh S, Ashrafizadeh H, Mojaddami F, kord B (2017). A survey on knowledge level of the nurses about hospital triage. J Nurs Educ (JNE).

[2] Kathleen C (2016). Crowding in the Emergency Department. J Emerg Nurs.

[3] Love RA, Murphy JA, Lietz TE (2012). The effectiveness of a provider in triage in the emergency department: a quality improvement initiative to improve patient flow. Adv Emerg Nurs J.

[4] Ajani K (2012). Triage; a literature review of key concepts. J Pak Med Assoc.

[5] Van der Linden MC, Meester BE (2016). Van der Linden N. Emergency department crowding affects triage processes. Int Emerg Nurs.

[6] Kerie S, Tilahun A, Mandesh A (2018). Triage skill and associated factors among emergency nurses in Addis Ababa, Ethiopia 2017: a cross-sectional study. BMC Res Notes.

[7] Vatnøy TK, Fossum M, Smith N (2013). Triage assessment of registered nurses in the emergency department. Int Emerg Nurs.

[8] Phukubye TA, Mbombi MO, Mothiba TM (2019). Knowledge and practices of triage amongst nurses working in the emergency departments of rural hospitals in Limpopo Province. Open Public Health J.

[9] Christian M (2015). To score or not to score during triage in the emergency department. Intensive Care Med.

[10] Lin D (2013). Predictors of admission to hospital of patients triaged as nonurgent using the canadian triage and acuity scale. CJEM.

[11] Javadi N, Rostamnia L, Raznahan R, Ghanbari V (2021). Triage training in iran from 2010 to 2020: a systematic review on educational intervention studies. Iran J Nurs Midwifery Res.

[12] Bijani M, Torabizadeh C, Rakhshan M, Fararouei M (2018). Professional capability in triage nurses in emergency department: a qualitative study. Revista Latinoam de Hipertensión.

[13] Grossmann FF, Zumbrunn T, Frauchiger A (2012). At risk of under triage? Testing the performance and accuracy of the emergency severity index in older emergency department patients. Ann Emerg Med.

[14] Phukubye TA, Mbombi MO, Mothiba TM (2021). Strategies to Enhance Knowledge and practical skills of Triage amongst Nurses Working in the Emergency Departments of Rural Hospitals in South Africa. Int J Environ Res Public Health.

[15] Duko B, Geja E, Oltaye Z, Belayneh F, Kedir A, Gebire M (2019). Triage knowledge and skills among nurses in emergency units of Specialized Hospital in Hawassa, Ethiopia: cross sectional study. BMC Res Notes.

[16] Aslanoglu A, Ayyildiz M. Triage knowledge levels of nurses in Turkey: A study in state hospitals in Samsun. New Trends and Issues Proceedings on Advances in Pure and Applied Sciences. 2021(13):150–161.

[17] Bista s. mukhia s, rai l, chaudhary tk. Knowledge on triage management among nurses in a tertiary level hospital of kathmandu.Asian journal of advances in medical science. 2022:57–65.

[18] Twagirayezu I, Busisiwe B, Cishahayo EU (2021). Knowledge and skills on triage among nurses working in emergency departments in Referral Hospitals in Rwanda. Rwanda J Med Health Sci.

[19] Mirhaghi AH, Roudbari M (2011). A survey on knowledge level of the nurses about hospital triage. Q Iran J Crit Care Nurs.

[20] Bijani M, Rakhshan M, Fararouei M, Torabizadeh C (2020). Development and psychometric assessment of the triage nurses’professional capability questionnaire in the emergency department. BMC Nurs.

[21] Bijani M, Khaleghi A. Challenges and Barriers Affecting the Quality of Triage in Emergency Departments: A Qualitative Study.Galen Med J.2019:1–7. 10.31661/gmj.v8i0.1619 PMC8344134 34466538

[22] Najafi Z, Abbaszadeh A, Vaezi H, Rassouli M, Mirhaghi A, Zohari Anboohi S (2021). Hospital Triage Standards: a qualitative study and content analysis based on experts’ Experiences in Iran. Front Emerg Med.

[23] Ayre A, Scally AJ (2014). Critical values for Lawshe’s content validity ratio: revisiting the original methods of calculation. Meas Evaluation Couns Dev.

[24] Al-Rawee RY, Abdulghani MF, AlSalih AA-RM, Mohammed EH, Tawfeeq BA-G, Knowledge (2022). Attitude and practice of nursing staff toward working at Emergency Unit. Annals of the College of Medicine Mosul.

[25] Haghdoust. z, safavi m, yahyavi h. Effect of triage education on knowledge, attitude and practice of nurses in poursina educational and therapeutic emergency center in rasht. J Holist Nurs Midwifery. 2010;20(2): 14–21.

[26] Rahmati H, Azmoon M, Kalantari Meibodi M, Zare N (2013). Effects of Triage Education on Knowledge, Practice and qualitative index of Emergency Room Staff: a quasi-interventional study. Bull Emerg Trauma.

[27] Kantar L, Alexander R (2012). Integration of Clinical Judgment in the nursing curriculum: Challenges and Perspectives. J Nurs Educ.

[28] Eghbali S, Najafi M, Emergency Nurses Job Satisfaction Prediction Model (2020). Personality traits, resilience, emotional expression and ambiguity tolerance. J Mol Biol Res.

[29] Lin C-C, Liang H-F, Han C-Y, Chen L-C, Hsieh C-L (2019). Professional resilience among nurses working in an overcrowded emergency department in Taiwan. Int Emerg Nurs.

[30] Ahmadi B, Mosadeghrad AM, Karami B (2019). Effectiveness of resilience education on quality of working life among nursing personnel: a randomized controlled study. Payesh.

[31] Aloyce R, Leshabari S, Brysiewicz P (2014). Assessment of knowledge and skills of triage amongst nurses working in the emergency centres in Dar es Salaam, Tanzania. Afr J Emerg Med.

[32] Widgren BR, Jourak M (2012). Medical Emergency Triage and Treatment System (METTS): a new protocol in primary triage and secondary priority decision in emergency medicine. J Emerg Med.

