# Correction: Investigating the professional capability of triage nurses in the emergency department and its determinants: a multicenter cross-sectional study in Iran

**DOI:** 10.1186/s12873-023-00835-5

**Published:** 2023-05-29

**Authors:** Maryam Aghabarary, Zahra Pourghaedi, Mostafa Bijani

**Affiliations:** 1 Social Determinants of Health Research Center, Alborz University of Medical Sciences, Karaj, Iran; 2 Student Research Committee, Alborz University of Medical Sciences, Karaj, Iran; 3 Department of Medical Surgical Nursing, School of Nursing, Fasa University of Medical Sciences, Fasa, Iran

BMC Emergency Medicine (2023) 23:38

10.1186/s12873-023-00809-7

The original article [1] contained erroneous text in the authorship which has since been removed.
