# Supplementary material for: Investigating the professional capability of triage nurses in the emergency department and its determinants: a multicenter cross-sectional study in Iran
Source: BMC Emerg Med. 2023 Apr 1;23:38. doi: 10.1186/s12873-023-00809-7 (PMC10068142; doi:10.1186/s12873-023-00809-7)
Supplement: Supplementary file 1 — Supplementary Material 1 [file 12873_2023_809_MOESM1_ESM.pdf]

Supplementary file 1: Demographic characteristics of participants (n=580)

**Characteristic**		**N (%)**
**Sex**	Female	342 (59%)
	Male	238 (41%)
**Marital status**	Married	341 (58.8%)
	Single	237 (41.2%)
**Education level**	BSc in nursing	550 (94.8%)
	MSc in nursing	30 (5.2%)
**Shift**	Morning	8 (1.4%)
	Morning and evening	7 (1.2%)
	Rotating	565 (97.4%)
**Hospital name**	Namazi	204 (35.2%)
	Faghihi (Sa'adi)	132 (22.8%)
	Gerash	23 (4%)
	Larestan	30 (5.2%)
	Darab	68 (11.7%)
	Jahrom	61 (10.5%)
	Fasa	62 (10.7%)
**Interest in working as nurse triage**	Low	91 (15.7%)
	Moderate	452 (77.9%)
	High	37 (6.4%)
	**Mean (SD)**	**Range**
**Age (years)**	31.36 ± (5.02)	22-47
**Work experience as nurse (years)**	7.97 ± (5.39)	0.5-29
**Work experience as emergency nurse (years)**	6.68 ± (4.71)	0.5-26
**Work experience as triage nurse (years)**	4.95 ± (4.25)	0.5-24
